# The Sovereigns of England

**Published:** 1878-11

**Authors:** 


### THE SOVEREIGNS OF ENGLAND.

The following list of the sovereigns of England, with the terms of their individual reign, will be found convenient for reference :

First William and Norman ; then William his son ;
Henry, Stephen and Henry ; then Richard and John ;
Next Henry the third ; Edwards, one, two and three ;
And again, after Richard, three Henrys we see.
Two Edwards, third Richard, if rightly I guess ;
Two Henrys, sixth Edward, Queen Mary, Queen Bess.
Then Jamie the Scotchman ; then Charles, whom they slew,
Yet received, after Cromwell, another Charles too.
Next Jamie the second ascended the throne,
Then William and Mary together came on ;
Till Anne, the Georges and William all past,
God sent us Victoria—may she long be the last.

| | |
|---|---|
| William I | 1066 to 1087 |
| William II | 1087 — 1100 |
| Henry I | 1100 — 1135 |
| Stephen | 1135 — 1154 |
| Henry II | 1154 — 1189 |
| Richard I | 1189 — 1199 |
| John | 1199 — 1216 |
| Henry III | 1216 — 1272 |
| Edward I | 1272 — 1307 |
| Edward II | 1307 — 1327 |
| Edward III | 1327 — 1377 |
| Richard II | 1377 — 1399 |
| Henry IV | 1399 — 1413 |
| Henry V | 1413 — 1422 |
| Henry VI | 1422 — 1461 |
| Edward IV | 1461 — 1483 |
| Edward V | 1483 |
| Richard III | 1483 — 1485 |
| Henry VII | 1485 — 1509 |
| Henry VIII | 1509 — 1547 |
| Edward VI | 1547 — 1553 |
| Mary I | 1553 — 1558 |
| Elizabeth | 1558 — 1603 |
| James I | 1603 — 1625 |
| Charles I | 1625 — 1649 |
| (Commonwealth) | 1649 — 1660 |
| Charles II | 1660 — 1685 |
| James II | 1685 — 1689 |
| William III and Mary II | 1689 — 1694 |
| William III, alone | 1694 — 1702 |
| Anne | 1702 — 1714 |
| George I | 1714 — 1727 |
| George II | 1727 — 1760 |
| George III | 1760 — 1820 |
| George IV | 1820 — 1830 |
| William IV | 1830 — 1837 |
| VICTORIA | 1837. |

